# Diagnosis, treatment, and work impact of iron deficiency anemia in a Portuguese urban community

**DOI:** 10.1097/j.pbj.0000000000000064

**Published:** 2020-07-17

**Authors:** Pedro Norton, Natália Araújo, Paulo Pinho, Joana Costa Gomes, Carla Silva, Céline Gama, Manuel Barbosa, Pedro Ferreira, Sara Cunha, Sara Moreira, Sílvia Martins, Sofia Faria, Sophie Sousa, Nuno Figueiras Alves, Nuno Lunet

**Affiliations:** a Occupational Health Service, S. João Hospital Center; b EPIUnit – Publick Health Institut, University of Porto, Rua das Taipas; c Department of Public Health and Forensic Sciences and Medical Education, University of Porto Medical School, Porto; d Health Centers Group of Matosinhos, Matosinhos, Portugal.

**Keywords:** absenteeism, anemia, iron compounds/therapeutic use, iron deficiency

## Abstract

**Introduction:**

Little is known about iron deficiency anemia (IDA)'s treatment in Portugal. We aim to estimate the proportion of anemia, IDA, and iron deficiency without anemia; characterize the diagnostic procedures and prescription patterns; assess anemia's impact over work absenteeism, in a Local Health Unit.

**Material and methods:**

Cross-sectional study that evaluated complete blood counts, iron-containing prescriptions, comorbidities, economic failure, and disability certificates issued in 2015 at the Local Health Unit.

**Results:**

We evaluated 62,794 complete blood count. The proportion of anemia was 16.5%, higher in patients with economic failure, pregnant women, and patients with congestive heart failure. Of the patients with anemia 87.8% had not serum iron and/or ferritin dosing, and of those with serum iron/ferritin levels tested 50.6% had IDA. IDA was higher in pregnant women, women aged ≥15 years and in patients with congestive heart failure. Approximately 56.2% of patients with IDA did not receive iron-containing medication, and in 38% of the cases the prescribed dose was subtherapeutic. Of the total iron prescriptions 44.1% were association therapies. Anemia accounted for 5.2% of the disability certificates issued in 2015 (1749 workdays lost).

**Discussion:**

Most patients with anemia are not being adequately evaluated and a major proportion does not undergo treatment or has subtherapeutic doses of iron. These results may explain the anemia's impact on work capacity.

**Conclusion:**

This is one of the largest studies on anemia in Portugal. An effort to adapt to the established recommendations is urged, to minimize the consequences of this disease.

## Introduction

The diagnosis of anemia in men is based on a hemoglobin of <13 g/dL; in women, it is <12 g/dL.^1^ Iron-deficiency anemia (IDA) is a diminished red blood cell production due to low iron stores in the body.^1^,^2^ Iron deficiency (ID) is not the only cause of anemia, but it usually is its most common cause, accounting for approximately half the anemia cases worldwide.^1^,^2^ In fact, the prevalence of anemia, defined by low hemoglobin or hematocrit, is commonly used to assess the severity of ID in a population.^3^

ID is the most common nutritional disorder and varies from iron depletion without anemia to IDA.^4^,^5^ Worldwide, the prevalence of IDA is 15% to 20% and varies greatly according to host factors: age, sex, physiological, pathological, environmental, and socioeconomic conditions.^1^,^6^ In the developed countries more than 500 million people are iron deficient.^7^,^8^,^9^

Anemia is associated with increased morbidity and mortality,^1^ and IDA may be the first presenting feature of an occult gastrointestinal malignancy.^10^,^11^,^12^

Anemia may exacerbate chronic diseases namely heart failure, and is associated with an increased risk of hospitalization in older adults.^13^,^14^ IDA during pregnancy has been associated with increased risk of preterm delivery, low birth weight, and maternal perinatal and young child mortality.^13^,^15^ From 1990 to 2010 IDA has only changed from 14th to 15th cause of global disability adjusted life years.^16^

A Portuguese survey conducted in 2015 in the adult population, showed a prevalence of anemia of 19.9%, and a prevalence of IDA of 5.8%.^17^ This survey revealed the surprising statistic that 84% of the individuals were not aware of being anemic and were not receiving any treatment. Despite that the impact of such finding on work absenteeism was not accessed, as much as 30% impairment of physical work capacity and performance is reported in iron-deficient men and women.^1^ Even without anemia, mild to moderate ID has adverse functional consequences.^18^ Mild ID without anemia could explain chronic fatigue and impaired cognitive performance.^1^ Iron supplementation improves progressive fatigue resistance.^19^,^20^

Although prevalence of ID is already known, the extent to which ID is effectively treated in the general population and the compliance with the national guidelines are not so well documented.^21^

This study aims to estimate the prevalence of anemia, IDA and ID without anemia among patients of Matosinhos Local Health Unit medical services (MLHU); to characterize the diagnostic procedures and prescription patterns regarding compliance with national guidelines, and to access anemia impact over work absenteeism.

## Methodology

### Study design

We conducted a cross-sectional study in MLHU which is a mixed unit composed by all public healthcare services (namely 1 hospital and 14 primary care centers) of Matosinhos, a county located at the north of Portugal with 172,366 patients. Between January 1st and December 31st of 2015 we accessed all blood counts analyzed in the MLHU Clinical Pathology Laboratory. We used the World Health Organization (WHO) criteria for diagnosing anemia.^1^ To characterize the diagnostic procedures according to the Portuguese Directorate-General of Health Guidelines, we evaluate the proportion of anemic patients with blood iron or ferritin levels accessed. The cut-off for ID was set for a serum iron <50 μg/dL or a serum ferritin <30 ng/mL.^5^

The study included all outpatient drugs used for the treatment of iron-deficiency anemia in MLHU (Table 1).

**Table 1:**
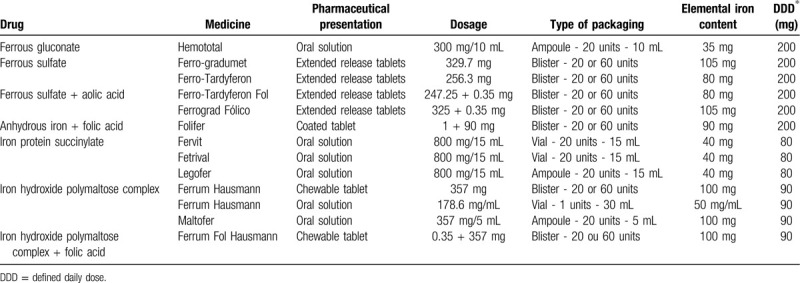
Defined daily dose of drugs with iron compounds and or associations of iron compounds with folic acid available in Portugal

The list of these medications was obtained by consultation of the drug database, Informed of the National Institute of Pharmacy and Medicine, with the filter Therapeutic Classification “4.1.1. iron compounds.”^22^

As the Portuguese General Directorate of Health recommends a daily dose of 100 to 200 mg of elemental iron for treatment of ID, we set a prescribed daily dose (PDD) to 100 mg of elemental iron for all medications included in this study.^5^

The pregnant women and the patients with congestive heart failure (CHF) were identified from the electronic clinical database using the International Classification of Primary Care, Second Edition codes K77 and W78.^23^ The patients with economic failure were identified using the National Information System of the Primary Health Care. This administrative economic status is granted to patients who integrate a household whose average monthly income, divided the number of people who are responsible for the direction of the household (taxpayers in terms of tax return), is ≤€628.83 (1.5 times the Social Support Index).^24^

### Statistical analysis

The association of anemia with sex, pregnancy, age, economic failure, and CHF was analyzed with the calculation of odds ratio using logistic regression models. The analysis was performed in STATA 11.0 (StataCorporation, College Station, TX).

The study protocol was approved by the MLHU Ethics Committee.

## Results

We evaluated 62,794 complete blood counts (CBC), ranging 36.4% of the 172,366 MLHU patients. The proportion of anemia found was 16.5% (n = 10,387), higher in patients with economic failure (18.7%), pregnant women (21.0%), and patients with CHF (47.4%) (Fig. 1 ; Table 2).

**Figure 1:**
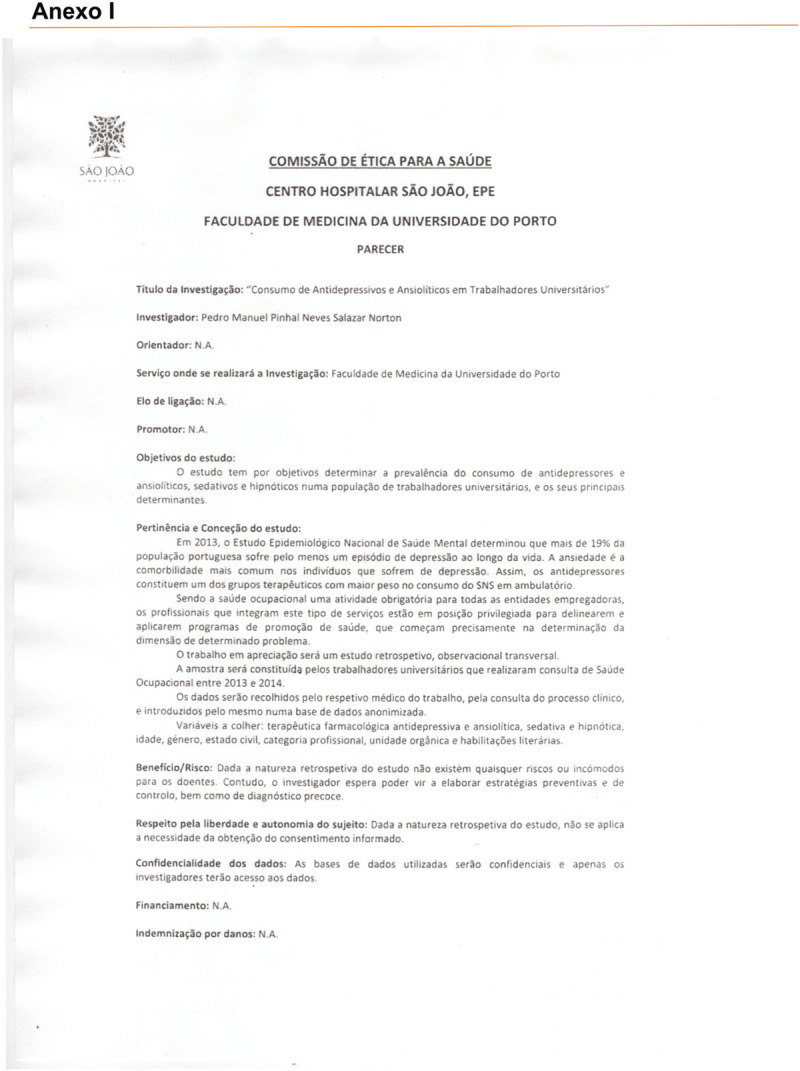
Schematic representation of results. CBC = complete blood count, ID = iron deficiency.

**Figure 1 (Continued):**
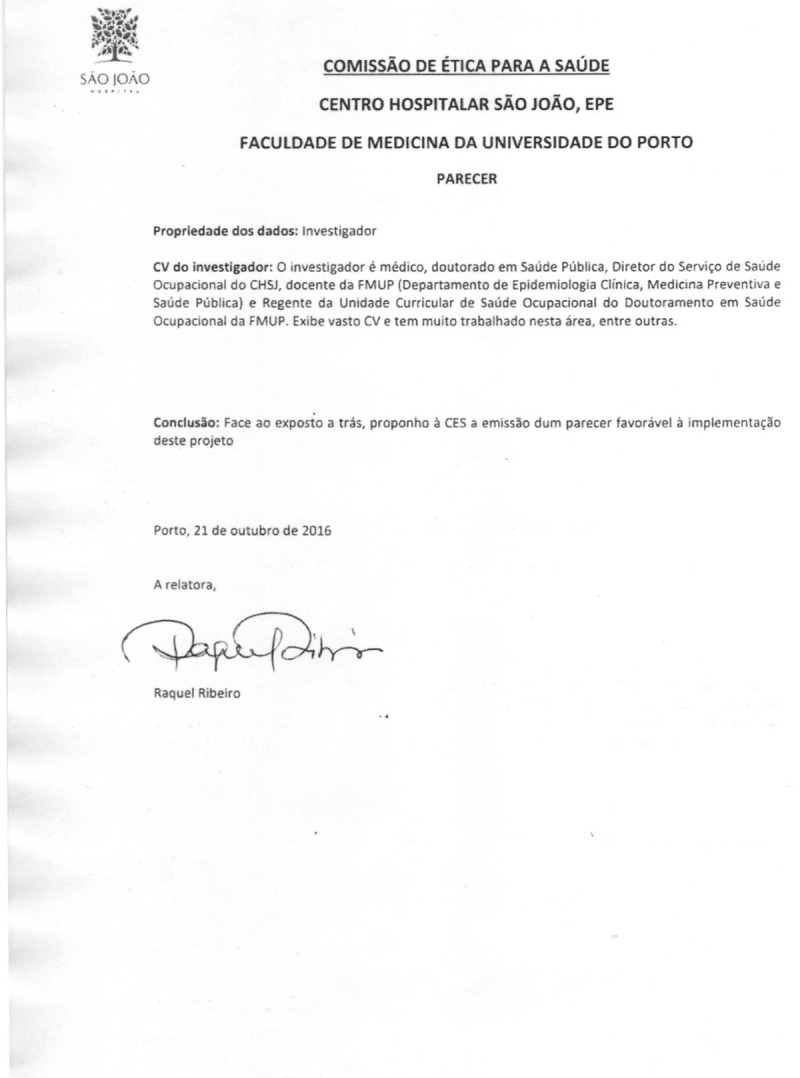
Schematic representation of results. CBC = complete blood count, ID = iron deficiency.

**Figure 1 (Continued):**
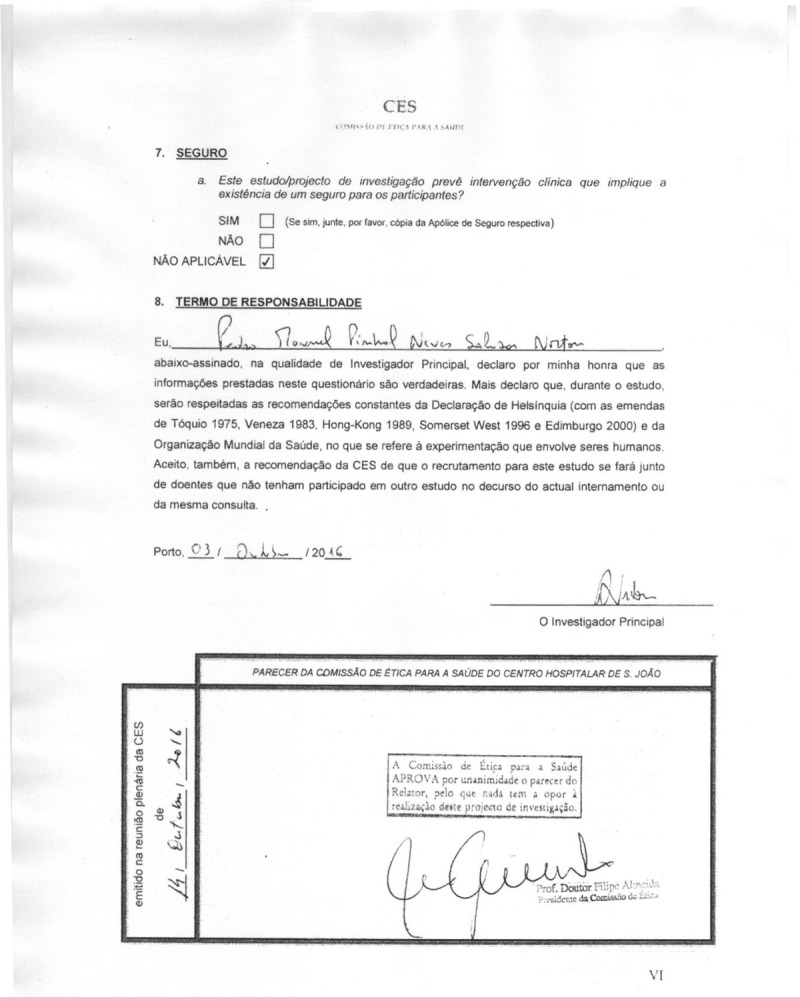
Schematic representation of results. CBC = complete blood count, ID = iron deficiency.

**Table 2:**
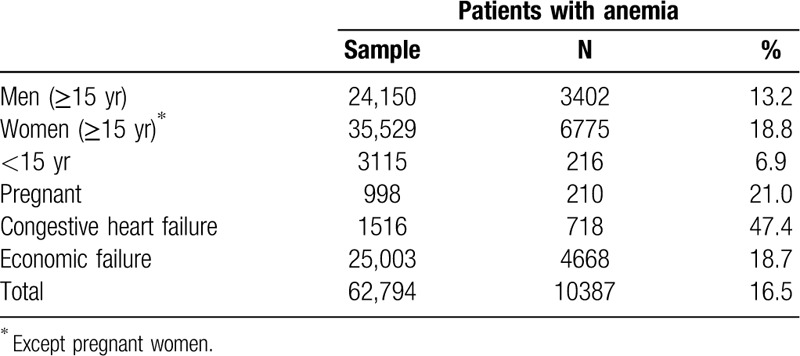
Proportion of Matosinhos Local Health Unit patients with anemia

The hemoglobin levels were less than 10 mg/dL in 18.4% (n = 1915) of the anemic patients.

Of the 10,387 patients with analytically proven anemia, 87.8% (n = 9121) had no serum iron and/or ferritin quantified during the study period.

Of the 1266 patients with anemia and serum iron/ferritin levels tested, 50.6% (n = 640) had IDA.

Of the 2291 patients with normal hemoglobin value and an iron/ferritin assay, 307 (13.4%) had ID.

The proportion of patients with IDA was particularly high in pregnant women (62.5%), women aged 15 years or older (55.8%), and patients with CHF (47.2%) (Table 3).

**Table 3:**
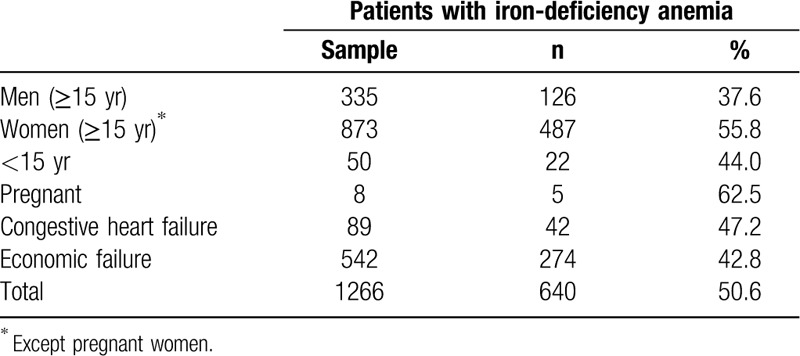
Proportion of Matosinhos Local Health Unit patients with iron-deficiency anemia

In the logistic regression analysis, the risk of anemia was significantly higher in women [adjusted odds ratio (OR) 1.56, 95% confidence interval (CI) 1.20–2.04] (Table 4).

**Table 4:**
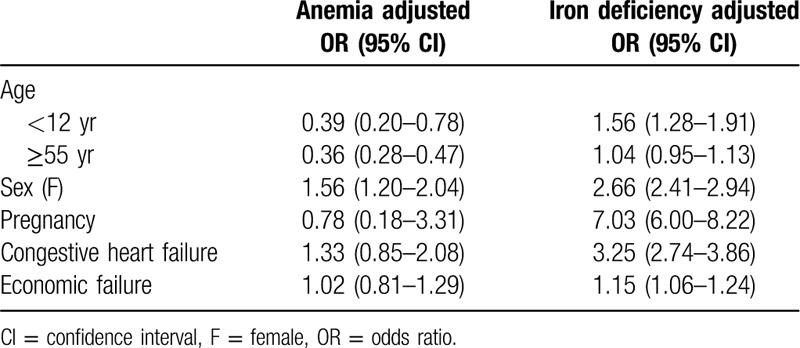
Odds ratio of anemia and iron deficiency using logistic regression model

The risk of ID was higher in women (adjusted OR 2.66, 95% CI 2.41–2.94), pregnant women (adjusted OR 7.03, 95% CI 6.0–8.22), patients with <12 years of age (adjusted OR 1.56, 95% CI 1.28–1.91), patients with economic deficiency (adjusted OR 1.15, 95% CI 1.06–1.24), and patients with CHF (adjusted OR 3.25, 95% CI 2.74–3.86).

During the study period iron-containing medication was prescribed to 43.8% (n = 280) of patients with analytically proven IDA and to 9.1% (n = 28) of patients with ID without anemia. A total of 314,358 PDDs of oral iron were prescribed to MLHU patients, excluding pregnant women during the year 2015. The distribution of PPDs was 55.9% for monotherapy and 44.1% for therapeutic associations containing oral iron (Table 5).

**Table 5:**
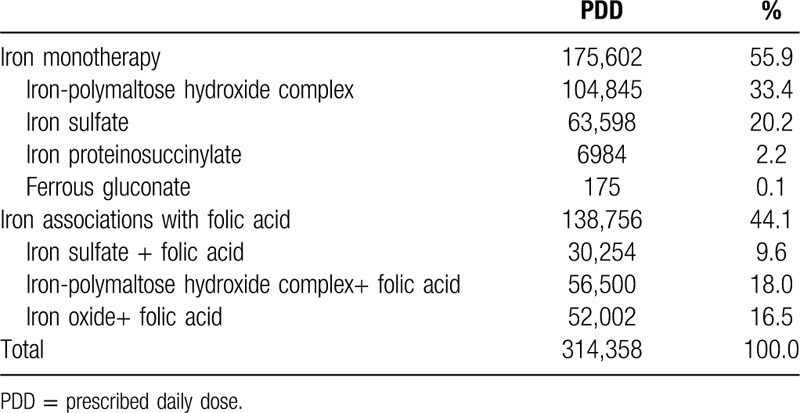
Iron-containing medication prescribed (except pregnant women)

With respect to monotherapy, the PDD distribution by active substance was 33.4% for iron-polymaltose hydroxide complex, 20.2% for iron sulfate, 2.1% for iron protein succinylate, and 0.1% for iron gluconate. The treatment was carried out with a daily dose of elemental iron of <100 mg in 38% of cases (regardless of whether it was prescribed as monotherapy or fixed combination).

In 2015, of the patients who performed at least 1 CBC at MLHU, 1278 had a Disability Certificate for Work as a result of natural disease. Of these, absence to work was specifically motivated by anemia diagnosis in 67 (5.2%), corresponding to a total of 1749 workdays lost (4.79 years), with a median of 12 days of absence for each Disability Certificate for Work issued. The median number of workdays lost was similar (n = 12) in patients with anemia who were and were not medicated, but was lower in patients with IDA (n = 10) either medicated (n = 11) or unmedicated (n = 4).

## Discussion

This is one of the largest anemia studies conducted in Portugal, evaluating 62,794 blood counts, covering 36.4% of the 172,366 patients enrolled in the MLHU. The proportion of anemia found was 16.5%, a figure higher than that estimated by the WHO for Portugal (15%),^24^ but lower than what was found in another Portuguese study (19.9%), which only evaluated patients aged 18 years or more.^17^

It should be noted that the results reflect the proportion of patients with anemia who underwent a blood count during the study period, rather than the prevalence of anemia in the municipality of Matosinhos. However, the MLHU is an institution that integrates primary and secondary healthcares, internalizing some complementary means of diagnosis, so most of the CBC and biochemistry with iron kinetics prescribed in this local health unit are analyzed at the MLHU Clinical Pathology Laboratory. As such, this study has analyzed not only hospital but also primary health care prescriptions.

The General Directorate of Health recommends ID screening, regardless the clinical condition, for pregnant women, infants, and preschool children (6–12 months old) with poor social-economic conditions, institutionalized elderly, at hospital admission, and at preoperative period.^25^ The Centers for Disease Control and Prevention recommends periodic screening among high-risk populations of infants and preschool children, pregnant women, and nonpregnant women of childbearing age, every 5 to 10 years starting in adolescence.^15^

The most common method of screening ID involves determining the prevalence of anemia by measuring blood hemoglobin or hematocrit levels.^1^ Hematocrit measurement is an acceptable and recommended method for anemia determination, but has no advantage compared to hemoglobin measurement.^1^ A major limitation of hemoglobin or hematocrit measurements is that levels change only when they are very low at the outset, and when ID is already severe.^26^ In resource-adequate situations, the usual practice involves the use of further, specific, and more sensitive tests for individual assessment such as serum iron or serum ferritin,^27^ although the biological variability of serum iron limits its utility for daily practice.^28^,^29^

Of the 10,387 patients with analytically proven anemia, the majority (87.8%) had no serum iron and/or ferritin quantified during the study period, which may reflect an insufficient diagnostic investigation of the anemia cases found in all subgroups analyzed.

As expected, the proportion of patients with IDA was particularly high in pregnant women and patients with CHF. It is also noted that, in addition to the increased IDA risk in CHF, these patients were 3.25 times more likely to have ID (OR 3.25; 95% CI 2.74–3.86). As such, it is fundamental to systematically determine serum iron or ferritin in addition to the CBC in this patient subset.

According to the Clinical Guideline of the General Directorate of Health's regarding the treatment of ID in the adult, oral iron is the first-line option as its administration is simple, physiological, economical, and safe.^5^ There are multiple formulations on the market, and a daily dose of 100 to 200 mg of elemental iron is recommended. Nevertheless, in this study 56.2% of patients with IDA did not receive iron-containing medication, and in 38% of cases the prescribed dose was subtherapeutic (<100 mg/day). The absence or subtherapy may be associated with the perpetuation of the disease and the symptoms. In the specific case of CHF, 47.2% of the patients had concomitant IDA. According to the Guidelines of the European Society of Cardiology,^30^ the diagnosis and treatment of IDA in CHF is particularly important since its nontreatment is associated with a worse prognosis. Iron supplementation should always be considered for the improvement of CHF symptoms, exercise capacity and quality of life when ferritin <100 μg/L or when ferritin 100 to 299 μg/L and transferrin saturation <20%. However, we found that iron medication was prescribed to only 42.9% of these patients.

Provided that the dosage allows reaching the recommended daily dose, all the iron active principles are valid options. Nevertheless, the treatment should be adapted to the patient tolerability. Of the formulations available in Portugal, there are only 2 active substances that allow to reach the dose of elemental iron recommended in only one daily intake (iron hydroxide polymaltose complex and ferrous sulfate), which may increase adherence and therapeutic maintenance. However, ferrous sulfate is only available in sustained release forms and therefore its use should not be considered as the first line.^31^ Nonetheless, 20.2% of the packages prescribed in MLHU during the study period are sustained release formulations.

In IDA treatment, the association of iron with folic acid is not advisable since the patient's response may not be easily interpreted.^32^ Despite this recommendation, 44.1% of the total iron prescriptions corresponded to association therapies, excluding prescriptions for pregnant women. This therapeutic strategy does not improve the treatment effectiveness and may even increase the frequency of adverse effects.^32^ Moreover, from the economic point of view, the therapeutic associations adds a considerable cost to the patient and to the National Health Service because they have a higher retail price than that of monotherapy.

Therefore, an effort is required to adapt to the national and international recommendations, encouraging the training and continuous evaluation of the indicators evidenced in this study to minimize the consequences of this pathology. In addition, it is estimated that 10% to 20% of patients discontinue oral iron therapy due to side effects.^33^ Intolerance to oral iron will certainly be one of the explanations for the low proportion of patients treated in this study. In this case, the alternative is intravenous injection iron therapy, which is of exclusive hospital use in Portugal.

Therefore, patients must first be referred to the hospital outpatient consultation and then referred to the Day Hospital for treatment. It may take several months from the request of the Primary Care physician to the effective treatment in the Day Hospital, with the risk of clinical worsening of the patient. The establishment of protocols for referral of patients directly to hospital care, namely to the Day Hospital for treatment with intravenous iron in cases of intolerance or inefficacy of oral iron, would be an important way to increase the proportion of patients treated. Suspected infection may also explain some cases of undertreatment with iron, particularly in the hospital setting. However those cases are not significant for the conclusions of this study.

The differential diagnosis with anemia of inflammation is challenging. The soluble transferrin receptor could be useful, although the cost of its evaluation makes difficult the large-scale implementation. We also think that diet recommendations are often and erroneously used instead of iron supplements to treat low-grade anemia. This can contribute to the undertreatment. Bioavailability, especially of nonheme iron in anemic patients is low. It is difficult to achieve the intake of recommended daily dose (at least 100 mg) with diet alone.

Finally, it was evaluated the emission of Disability Certificates for Work by the patients with diagnosis of anemia in the study period. Anemia accounted for 5.2% of the disability certificates issued, demonstrating an important impact in the active population. The main reason was fatigue, which affected the absenteeism. The median number of workdays lost was similar in patients with anemia who were and were not medicated, but was lower in patients with IDA (either medicated or unmedicated). This probably reflects a greater severity of the disease rather than a better control of the hemoglobin value in the medicated group.

## Conclusion

The proportion of anemia was similar to that described by WHO for developed countries. The risk of IDA was particularly high in pregnant women, women younger than 15 years of age, and patients with CHF. Most patients with anemia were not being evaluated for iron kinetics. A large proportion of patients did not undergo any treatment or were undergoing subtherapeutic doses of oral iron. These results may explain the strong impact that anemia had on work capacity, accounting for 5.2% of all Work Disability Certificates issued in 2015 at MLHU.

## Acknowledgments

None.

## Conflicts of interest

The authors declare no conflicts of interest.

## References

[1] World Health Organization. Iron Deficiency Anaemia: Assessment, Prevention, and Control. A Guide for Programme Managers. Geneva: World Health Organization; 2001.

[2] Johnson-Wimbley TD Graham DY Diagnosis and management of iron deficiency anemia in the 21st century. Therap Adv Gastroenterol. 2011;4:177–184. 10.1177/1756283X11398736 PMC3105608 21694802

[3] Stoltzfus RJ Dreyfuss ML Guidelines for the Use of Iron Supplements to Prevent and Treat Iron Deficiency Anemia. International Nutritional Anemia Consultative Group (INACG). Geneva: World Health Organization; 1998.

[4] Grosbois B Decaux O Cador B Cazalets C Jego P Human iron deficiency [in French]. Bull Acad Natl Med. 2005;189:1649–1663. 16737092

[5] Direção Geral de Saúde. Abordagem, Diagnóstico e Tratamento da Ferropénia no Adulto. Lisboa; Direção Geral de Saúde, 16/01/2015. Report No.: Norma 030/2013.

[6] DeMaeyer E Adiels-Tegman M The prevalence of anaemia in the world. World Health Stat Q. 1985;38:302–316. 3878044

[7] Cook JD Iron-deficiency anaemia. Baillieres Clin Haematol. 1994;7:787–804. 7881154 10.1016/s0950-3536(05)80124-6

[8] Looker AC Dallman PR Carroll MD Gunter EW Johnson CL Prevalence of iron deficiency in the United States. JAMA. 1997;277:973–976. 9091669 10.1001/jama.1997.03540360041028

[9] Hercberg S Preziosi P Galan P Iron deficiency in Europe. Public Health Nutr. 2001;4:537–545. 11683548 10.1079/phn2001139

[10] Rockey DC Cello JP Evaluation of the gastrointestinal tract in patients with iron-deficiency anemia. N Engl J Med. 1993;329:1691–1695. 8179652 10.1056/NEJM199312023292303

[11] McIntyre AS Long RG Prospective survey of investigations in outpatients referred with iron deficiency anaemia. Gut. 1993;34:1102–1107. 8174963 10.1136/gut.34.8.1102 PMC1374363

[12] Ioannou GN Rockey DC Bryson CL Iron deficiency and gastrointestinal malignancy: a population-based cohort study. Am J Med. 2002;113:276–280. 12361812 10.1016/s0002-9343(02)01214-7

[13] Clark SF Iron deficiency anemia. Nutr Clin Pract. 2008;23:128–141. 18390780 10.1177/0884533608314536

[14] Culleton BF Manns BJ Zhang J Tonelli M Klarenbach S Hemmelgarn BR Impact of anemia on hospitalization and mortality in older adults. Blood. 2006;107:3841–3846. 16403909 10.1182/blood-2005-10-4308

[15] Centers for Disease Control and Prevention Recommendations to prevent and control iron deficiency in the United States. MMWR Recomm Rep. 1998;47:1–29. 9563847

[16] Murray CJ Lopez AD Measuring the global burden of disease. N Engl J Med. 2013;369:448–457. 23902484 10.1056/NEJMra1201534

[17] Fonseca C Marques F Robalo Nunes A Belo A Brilhante D Cortez J Prevalence of anaemia and iron deficiency in Portugal: the EMPIRE study. Intern Med J. 2016;46:470–478. 26841337 10.1111/imj.13020

[18] Scrimshaw N Enwonwu C Functional significance of iron deficiency: an overview. Annual Nutrition Workshop Series. Nashville, TN: Meharry Medical College; 1990;1–13.

[19] Beutler E Larsh SE Gurney CW Iron therapy in chronically fatigued, nonanemic women: a double-blind study. Ann Intern Med. 1960;52:378–394. 13800263 10.7326/0003-4819-52-2-378

[20] Brutsaert TD Hernandez-Cordero S Rivera J Viola T Hughes G Haas JD Iron supplementation improves progressive fatigue resistance during dynamic knee extensor exercise in iron-depleted, nonanemic women. Am J Clin Nutr. 2003;77:441–448. 12540406 10.1093/ajcn/77.2.441

[21] Shander A Goodnough LT Javidroozi M Iron deficiency anemia—bridging the knowledge and practice gap. Transfus Med Rev. 2014;28:156–166. 24931617 10.1016/j.tmrv.2014.05.001

[22] INFARMED - Autoridade Nacional do Medicamento e Produtos de Saúde I.P. Infomed - base de dados de medicamentos de uso humano. Available at: http://app7.infarmed.pt/infomed/inicio.php. Accessed Janusary 23, 2016.

[23] WONCA, International Classification Committee (WICC) International Classification of Primary Care, Second Edition (ICPC-2). Oxford, UK: Oxford University Press; 1998.

[24] General Secretariat of the Ministry of Health. User fees - recognition of economic failure for payment exemption. Portal do Cidadão2016; Available at: https://www.portaldocidadao.pt/en/web/secretaria-geral-do-ministerio-da-saude/taxas-moderadoras-reconhecimento-de-insuficiencia-economica-para-isencao-de-pagamento. Accessed Janusary 23, 2016.

[25] Direção Geral da Saúde. Prescrição e Determinação do Hemograma. Lisboa: Direção Geral da Saúde,12/09/2013. Report No.: Norma 063/2011.

[26] Thomas DW Hinchliffe RF Briggs C Macdougall IC Littlewood T Cavill I British Committee for Standards in Haematology Guideline for the laboratory diagnosis of functional iron deficiency. Br J Haematol. 2013;161:639–648. 23573815 10.1111/bjh.12311

[27] Beutler E Hoffbrand AV Cook JD Iron deficiency and overload. Hematology Am Soc Hematol Educ Program. 2003;40–61. 14633776 10.1182/asheducation-2003.1.40

[28] Freedman M Sutin D Tallis R Fillit H Brocklehurst J Blood disorders and their management in old age. Brocklehurst's Textbook of Geriatric Medicine and Gerontology. 5th ed London: Churchill Livingstone; 1998;1247–1291.

[29] Markus HS Diagnostic investigations in the elderly: the clinical usefulness of serum iron and transferrin measurements in the diagnosis of iron deficiency anaemia. An audit. Br J Clin Pract. 1989;43:451–453. 2611108

[30] Ponikowski P Voors AA Anker SD 2016 ESC Guidelines for the diagnosis and treatment of acute and chronic heart failure: The Task Force for the diagnosis and treatment of acute and chronic heart failure of the European Society of Cardiology (ESC). Developed with the special contribution of the Heart Failure Association (HFA) of the ESC. Eur J Heart Fail. 2016;18:891–975. 27207191 10.1002/ejhf.592

[31] Norte ARS Anemia ferropénica: sugestões terapêuticas. Comissão de Farmácia e Terapêutica; Julho. 2015.

[32] Adamson JW Fauci AS Braunwald E Kasper DL Hauser SL Longo DL Jameson JL Iron deficiency and other hypoproliferative anemias. Harrison's Principles of Internal Medicine. 2008;McGraw Hill, 844–851.

[33] Little DR Ambulatory management of common forms of anemia. Am Fam Physician. 1999;59:1598–1604. 10193599

